# The determinants of C. difficile infection in long-term care facilities: a portrait of patient- and facility-level factors across 90 care regions in the veterans affairs health care system

**DOI:** 10.1186/2047-2994-4-S1-O36

**Published:** 2015-06-16

**Authors:** KA Brown, M Jones, F Adler, M Leecaster, K Nechodom, V Stevens, M Samore, J Mayer

**Affiliations:** 1 Epidemiology, University of Utah, Salt Lake City, UT, USA; 2 Mathematics, University of Utah, Salt Lake City, UT, USA; 3 University of Utah, Salt Lake City, UT, USA; 4 Pharmacy, University of Utah, Salt Lake City, UT, USA

## Introduction

*Clostridium difficile* infection (CDI) is an infectious diarrheal disease that is associated with antibiotic and healthcare exposures. Although individual-level risk factors have been extensively studied, the facility-level factors that drive CDI have not.

## Objectives

To study the determinants of CDI incidence across long term care (LTC) facilities, with a specific interest in the role importation of infectious patients from acute care (AC) facilities.

## Methods

We conducted a retrospective cohort study of CDI from 2006 through 2012 across Veterans Affairs local healthcare systems (HCS) where both AC and LTC patient censuses were above an average of 10 patients per day. Our outcome was LTC-onset *C. difficile* lab-identified event, defined as a case with onset ≥ 3 days after admission occurring at least 8 weeks from a previous positive test.

## Results

We identified 90 local HCS that met our inclusion criteria. The incidence of *C. difficile* infection in LTC facilities was 3.6 per 10,000 patient-days. In bivariate weighted linear regression analyses, the most important predictors of facility CDI incidence were importation (R2=0.63, p<0.001) and antibiotic prescribing (R2=0.58, p<0.001). Time-series analyses revealed that increases in *C. difficile* case importation from AC facilities preceded increases in CDI rates for a period of up to 8 weeks. Multi-level analyses, that included individual-level covariates, revealed that *C. difficile* importation and facility-level antibiotic use acted independently of resident age, direct antibiotic exposure and direct proton pump inhibitor use.

## Conclusion

This is the first study showing that importation of *C. difficile* cases from AC facilities and facility-level antibiotic use are principal drivers of CDI in LTC facilities. A regional approach, addressing rates in AC facilities, is needed to control CDI in LTC facilities.

## Disclosure of interest

None declared.

